# Simulated low-dose dark-field radiography for detection of COVID-19 pneumonia

**DOI:** 10.1371/journal.pone.0316104

**Published:** 2024-12-27

**Authors:** Rafael C. Schick, Henriette Bast, Manuela Frank, Theresa Urban, Thomas Koehler, Florian T. Gassert, Andreas P. Sauter, Bernhard Renger, Alexander A. Fingerle, Alexandra Karrer, Marcus R. Makowski, Daniela Pfeiffer, Franz Pfeiffer

**Affiliations:** 1 Chair of Biomedical Physics, Department of Physics & School of Natural Sciences, Technical University of Munich, Garching bei München, Germany; 2 Munich Institute of Biomedical Engineering, Technical University of Munich, Garching bei München, Germany; 3 Department of Diagnostic and Interventional Radiology, School of Medicine & Klinikum rechts der Isar, Technical University of Munich, München, Germany; 4 Philips Research, Hamburg, Germany; 5 Munich Institute for Advanced Study, Technical University of Munich, Garching bei München, Germany; 6 Kantonsspital Münsterlingen, Münsterlingen, Switzerland; Cameroon National Radiation Protection Agency, CAMEROON

## Abstract

**Background:**

Dark-field radiography has been proven to be a promising tool for the assessment of various lung diseases.

**Purpose:**

To evaluate the potential of dose reduction in dark-field chest radiography for the detection of the Coronavirus SARS-CoV-2 (COVID-19) pneumonia.

**Materials and methods:**

Patients aged at least 18 years with a medically indicated chest computed tomography scan (CT scan) were screened for participation in a prospective study between October 2018 and December 2020. Patients were included if they had a CO-RADS (COVID-19 Reporting and Data System) score ≥ 4 (COVID-19 group) or if they had no pathologic lung changes (controls). A total of 89 participants with a median age of 60 years (interquartile range 48 to 68 yrs.) were included in this study. Dark-field and attenuation-based radiographs were simultaneously obtained by using a prototype system for dark-field radiography. By modifying the image reconstruction algorithm, low-dose radiographs were simulated based on real participant images. The simulated radiographs corresponded to 50%, 25%, and 13% of the full dose (41.9 μSv, median value). Four experienced radiologists served as blinded readers assessing both image modalities, displayed side by side in random order. The presence of COVID-19-associated lung changes was rated on a scale from 1 to 6. The readers’ diagnostic performance was evaluated by analyzing the area under the receiver operating characteristic curves (AUC) using Obuchowski’s method. Also, the dark-field images were analyzed quantitatively by comparing the dark-field coefficients within and between the COVID-19 and the control group.

**Results:**

The readers’ diagnostic performance in the image evaluation, as described by the AUC value (where a value of 1 corresponds to perfect diagnostic accuracy), did not differ significantly between the full dose images (AUC = 0.86) and the simulated images at 50% (AUC = 0.86) and 25% of the full dose(AUC = 0.84) (p>0.050), but was slightly lower at 13% dose (AUC = 0.82) (p = 0.038). For all four radiation dose levels, the median dark-field coefficients within groups were identical but different significantly by 15% between the controls and the COVID-19 pneumonia group (p<0.001).

**Conclusion:**

Dark-field imaging can be used to diagnose the Coronavirus SARS-CoV-2 (COVID-19) pneumonia with a median dose of 10.5 μSv, which corresponds to 25% of the original dose used for dark-field chest imaging.

## 1. Introduction

Grating-based dark-field radiography is an X-ray imaging modality that simultaneously provides both a dark-field image and an attenuation image [1, 2]. The dark-field signal is caused by small angle scattering of the X-rays when passing through microstructure, such as pulmonary alveoli [2], whereas the attenuation image is generated by concomitant photo-electric absorption and Compton scattering of the X-rays.

Grating-based dark-field radiography has first been reported for imaging of lung tissue in preclinical studies [3–7], and has recently been translated to healthy humans [8] and to detect pulmonary diseases, such as emphysema [9, 10], combined pulmonary fibrosis and emphysema (CPFE) [11], lymphangioleiomyomatosis (LAM) [12] and most recently COVID-19 associated pneumonia [13]. In these pulmonary disease entities, the dark-field signal has been shown to be reduced due to a diminished number of tissue-air interfaces traversed by the X-rays, which in turn may be secondary to a destruction of the alveolar structure (e.g., emphysema) or to an inflammation of the alveoli (e.g., COVID-19).

As an emerging new imaging technology, the dose-setting for data acquisition in the initial clinical studies was accomplished by experiments performed with phantoms [14]. The effective dose currently employed in dark-field radiography for a posteroanterior image of the thorax is 35 μSv in a male reference person of 73kg weight and 173cm height [14, 15] and thus corresponds to only 0.5% to 2% of the average effective doses employed for computed tomography scan (CT scan) of the chest in clinical routine (standard CT: 7mSv [16], low-dose: 2mSv [17]). Compared to the average effective dose of 20μSv for conventional chest radiography [16], however, the effective dose in dark-field radiography is 1.75 times higher. Since dark-field X-ray imaging uses ionizing radiation, as does conventional radiography, the guiding principle of radiation safety, ALARA (As Low As Reasonably Achievable) [15] has to be put in focus for dark-field imaging, as well. Therefore, it is of clinical relevance to investigate if dose-reduced dark-field radiography is feasible without loss of diagnostic accuracy.

Therefore, the present study aims to evaluate the impact of simulated dose reductions in dark-field chest X-ray imaging on the detectability of COVID-19-induced changes of the lung by assessment of both reader ratings and dark-field coefficients as quantitative image parameters related to the functional integrity of pulmonary alveoli.

## 2. Materials & methods

For the present retrospective study, raw image data of 89 participants were employed that had been obtained from previous prospective monocentric studies at our institution, which had been approved by the Institutional Review Board of the Technical University of Munich (Ethics Commission of the Medical Faculty, Technical University of Munich, Germany; 587/ 16 S and 116/20 S) and the national radiation protection agency (Federal Office for Radiation Protection, Oberschleißheim, Germany, Z5-22462/2-2017-021 and Z5–22464/2020-047-G) and had been conducted in accordance with the Declaration of Helsinki (as revised in 2013). All participants had given prior informed written consent not only for participation in those previous studies but also for any future analysis of their image data. All participants had a minimum age of 18 years and had undergone a CT scan of the chest for a clinical indication at our hospital.

### 2.1. Study groups

**Fig 1:**
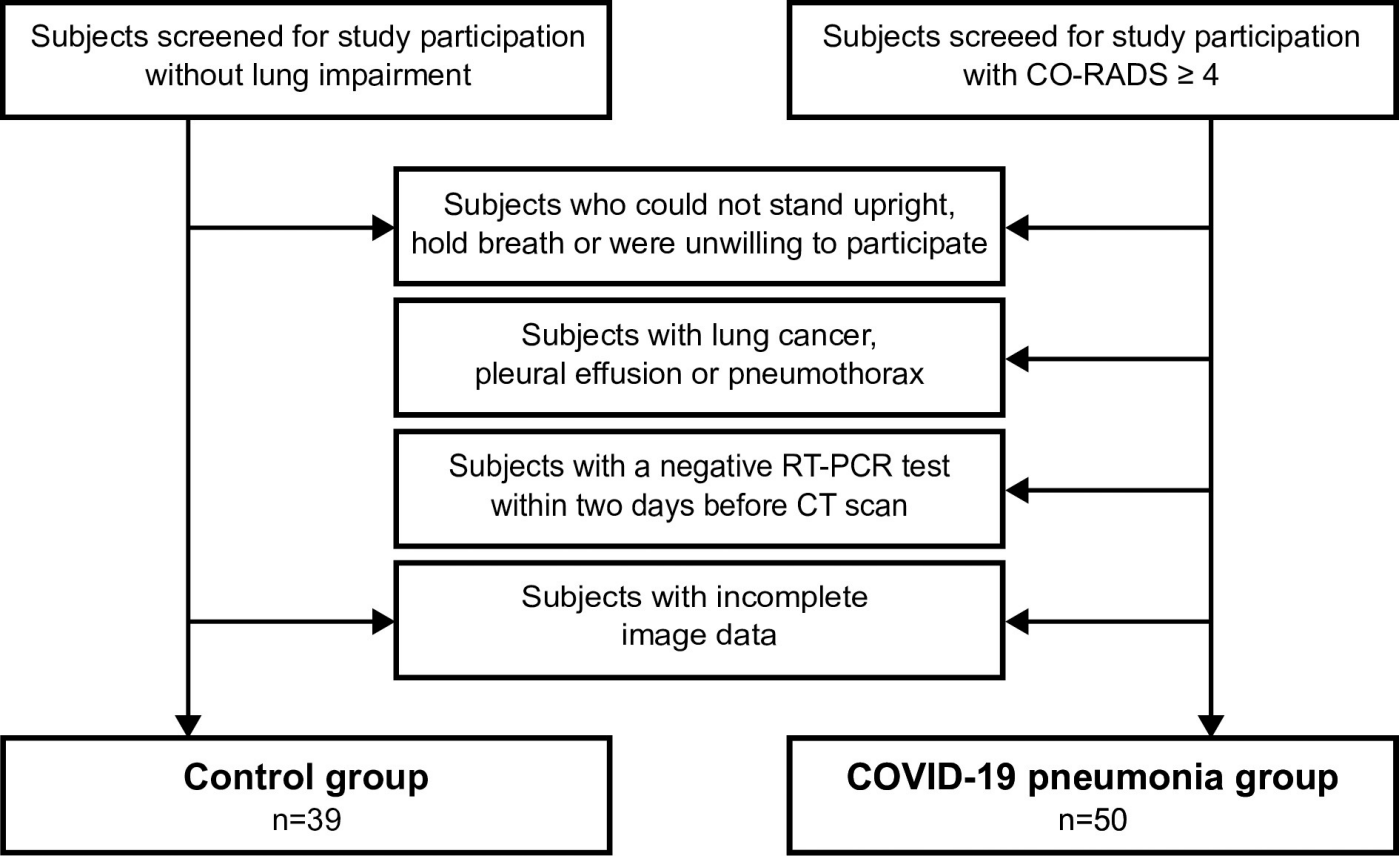
Protocol to determine study participants. Flow chart depicting the protocol for determining the study participants in the control group and the COVID-group. It shows the exclusion criteria for both groups of participants.

Image data of two groups of participants were included: a control group without any lung pathologies and a COVID-19 group. The inclusion procedure for both groups is described below (cf. Fig 1). Identification of potential study participants was performed on the basis of a screening of CT scans of the chest.

#### 2.1.1. Control group

Between October 2018 and January 2020, the CT scans of potential study participants for the control group were screened for the absence of pathologic lung changes by three radiologists (F. Gassert, A. Sauter, A. Fingerle) with 2, 6 and 12 years of experience in thoracic CT imaging. Furthermore, study participants had to consent and be able to stand upright without assistance. Also, participants who were pregnant or had pathological lung changes (such as cancer, pleural effusion, atelectasis, emphysema, infiltrates, ground glass opacities, and pneumothorax) were excluded. Participants with incomplete image data (e.g., an absent lateral dark-field image) were also excluded. The low-dose images of the control group were simulated on the basis of the full dose raw image data of 39 participants who had also been part of studies published in [8–10, 13, 14].

#### 2.1.2. COVID-19 group

Between May 2020 and December 2020, the CT scans of potential participants that were suspected to be infected with COVID-19 were screened for participation in the COVID-19 group. Two out of three radiologists (F. T. Gassert, A. Sauter, A. Fingerle) with 3, 7, and 13 years of experience in chest CT imaging performed a radiological consensus assessment on the level of suspicion for COVID-19 based on the CO-RADS criteria recently published by [18]. Only patients falling into the CO-RADS category 4 (high level of suspicion for COVID-19 based on CT imaging), 5 (very high level of suspicion for COVID-19), or 6 (clinically proven SARS-CoV-2 by RT-PCR test) were included in this COVID-19 study. Potential study participants had to consent and be able to stand upright without assistance. Potential participants were not included if they had a negative RT-PCR test within 2 days before the CT scan or if pregnancy, lung cancer, and pneumothorax were present. The full dose images of the 50 participants who formed the COVID-19 pneumonia group had previously been used to determine the suitability of dark-field radiography for assessing COVID-19 [13].

#### 2.1.3. CT imaging

CT imaging, which was employed for screening of study participants, was performed with routine clinical protocols at one of three CT scanners (Philips iCT, Siemens SOMATOM or Philips IQon Spectral CT). The CT scans were reconstructed with a lung-specific convolution kernel and 3 mm slice thickness.

### 2.2. Dark-field image acquisition

#### 2.2.1. Dark-field radiography

Dark-Field radiographs were generated at the Rechts der Isar Hospital of the Technical University of Munich, Germany employing a prototype X-ray scanning system which concomitantly provides attenuation radiographs during the scans of 7s duration as well. This prototype system uses a diagnostic X-ray tube operated at 70 kVp and a flat-panel detector with a field of view of 42cm x 42cm, see [9–11, 13, 14] for details. During image acquisition, each participant is standing in an upright position at full inspiration receiving median effective radiation doses of 41.9 μSv (posteroanterior) and 53.1 μSv (lateral), respectively.

#### 2.2.2. Simulated dose reduction

Both the dark-field and attenuation images were reconstructed from a series of raw intensity images by employing a phase retrieval algorithm specifically modified for our dark-field scanning system [19]. A reduction of the effective radiation dose was simulated by virtually narrowing the reconstruction area, a method that is also employed to correct motion artifacts in images generated by our prototype dark-field system [20]. In brief, the simulated dose reduction was accomplished by virtual stepwise cutting the grating area’s height of 140 px (42 cm x 6.5 cm) in half, i.e., to 70 px (50%), to 35 px (25%) and to 18 px (13%), respectively. In contrast to the motion artifact correction algorithm, where multiple reconstruction windows of fixed sizes are employed, for this study, a single variable sized reconstruction window (the size reduction corresponds proportionally to the dose reduction) was used to virtually reduce the radiation dose. By doing so, the number of data points available for fitting the phase stepping curve will decrease and gradually becomes insufficient during image reconstruction, thereby causing stripe artifacts. To alleviate this problem of insufficient phase coverage of the stepping curve during reconstruction of low-dose dark-field images, additional data points were included from neighboring pixels during reconstruction as previously described for the correction of motion artifacts [20]. As a trade-off, however, the effective resolution of the reconstructed images decreases.

### 2.3. Image evaluation and quantitative analysis

Image evaluation was performed between July 30, 2022 and December 20, 2022 by four radiologists (F. T. Gassert, A. Sauter, A. Fingerle and D. Pfeiffer) each with more than three years of experience in human dark-field imaging using a PACS system (Sectra IDS7, Linköping, Sweden) with authorized monitors. For the reader study, both posteroanterior attenuation and dark-field images of each participant were displayed side by side with window settings optimized for lung visualization for both modalities. Participants’ images were evaluated by the readers in randomized order and with the readers blinded to the participants’ group affiliation, personal data, clinical data and dose levels (100%, 50%, 25% or 13% of the full dose).

The presence of COVID-19-induced pneumonia was rated by all four readers individually on a scale from 1 to 6 (“1-surely not”, “2-very unlikely”, “3-unlikely”, “4-likely”, “5-very likely”, “6-surely”), see also [13]. General signal loss in dark-field images was considered as suggestive of COVID-19 pneumonia. Areas of focal signal loss were considered as highly suggestive of COVID-19 pneumonia and the combination of both features resulted in a definitive diagnosis of COVID-19 pneumonia.

For quantitative assessment, the dark-field coefficient (DFC) [8], as a measure correlated to the number of traversed tissue-air interfaces, was determined as the integrated dark-field signal over the lung area divided by the lung volume, estimated based on posteroanterior and lateral attenuation radiographs as described in [21].

### 2.4. Statistical analysis

If not stated otherwise, all values are presented as medians (interquartile range). A *p*-value of less than 0.050 was considered statistically significant.

Participants’ demographics of the COVID-19 group and the control group were tested for differences by Student’s t-test or the Chi-squared test, respectively.

The readers’ ratings for the likelihood of COVID-19 pneumonia were tested for differences between virtually reduced dose images and full dose images by use of the Wilcoxon signed-rank test, a non-parametric test for two dependent samples. When categorizing the rated likelihoods of COVID-19 pneumonia into the groups “COVID-19 pneumonia not present” (corresponding to ratings 1–3) or “COVID-19 pneumonia present” (corresponding to ratings 4–6) respectively, the differences between virtually reduced dose images and full dose images were tested by McNemar’s test, a test for paired nominal data. The inter-reader reliability was assessed by calculating Krippendorff’s alpha coefficient [22] for ordinal data, which was employed instead of kappa metrics, because it may be used for more than two readers and is much more reliable for a small sample size [23].

The diagnostic performance of the readers was evaluated by an analysis of the receiver operating characteristic (ROC) curve, employing Obuchowski’s method for nonparametric ROC analysis of correlated and clustered data [24]. The ROC curve is a plot of the true positive rate (sensitivity) against the true negative rate (1 minus specificity) and allows calculation of the area under the curve (AUC) with the AUC values (range: 0–1) representing a measure for the readers’ diagnostic performance with better performance being associated with higher AUC values.

The dark-field coefficients (DFC) at all dose levels were also tested for differences between the COVID-19 and the control group by the two-sided Mann-Whitney U test, a non-parametric test for independent samples.

Statistical analysis was performed by algorithms written in the programming languages Python, version 3.6.9, [25] and R, version 4.2.0, [26].

## 3. Results

### 3.1. Participant characteristics

Dark-field intensity data of a total of 89 participants (54 male, 35 female) were included in this study with a participants’ median age of 60 years (interquartile range 48 to 68 yrs.) and a median body weight of 77 kg (interquartile range 68 to 87 kg). No significant differences between the control group and participants with COVID-19 pneumonia were observed for the parameters sex, age, weight, and total lung volume (see Table 1).

**Table 1:** Participant parameters.

Parameter	All	Control	COVID-19	p-value
**Number of Participants**	89	39	50	
**Men/Women**	54/35	24/15	30/20	0.14
**Age (years)**	60 (48 to 68)	62 (54 to 70)	55 (47 to 67)	0.06
**Weight (kg)**	77 (68 to 87)	77 (68 to 87)	75 (67 to 89)	0.97
**Total Lung Volume (L)**	6.6 (5.5 to 7.7)	6.9 (5.8 to 7.7)	6.3 (5.3 to 7.8)	0.74

Data are reported as medians (interquartile range). P-values for differences of the participants’ parameters between the COVID-19 group and the control group are listed in the very right column. The threshold for statistical significance (p < .05) was not reached for any participant parameter.

### 3.2. Image appearance

**Fig 2:**
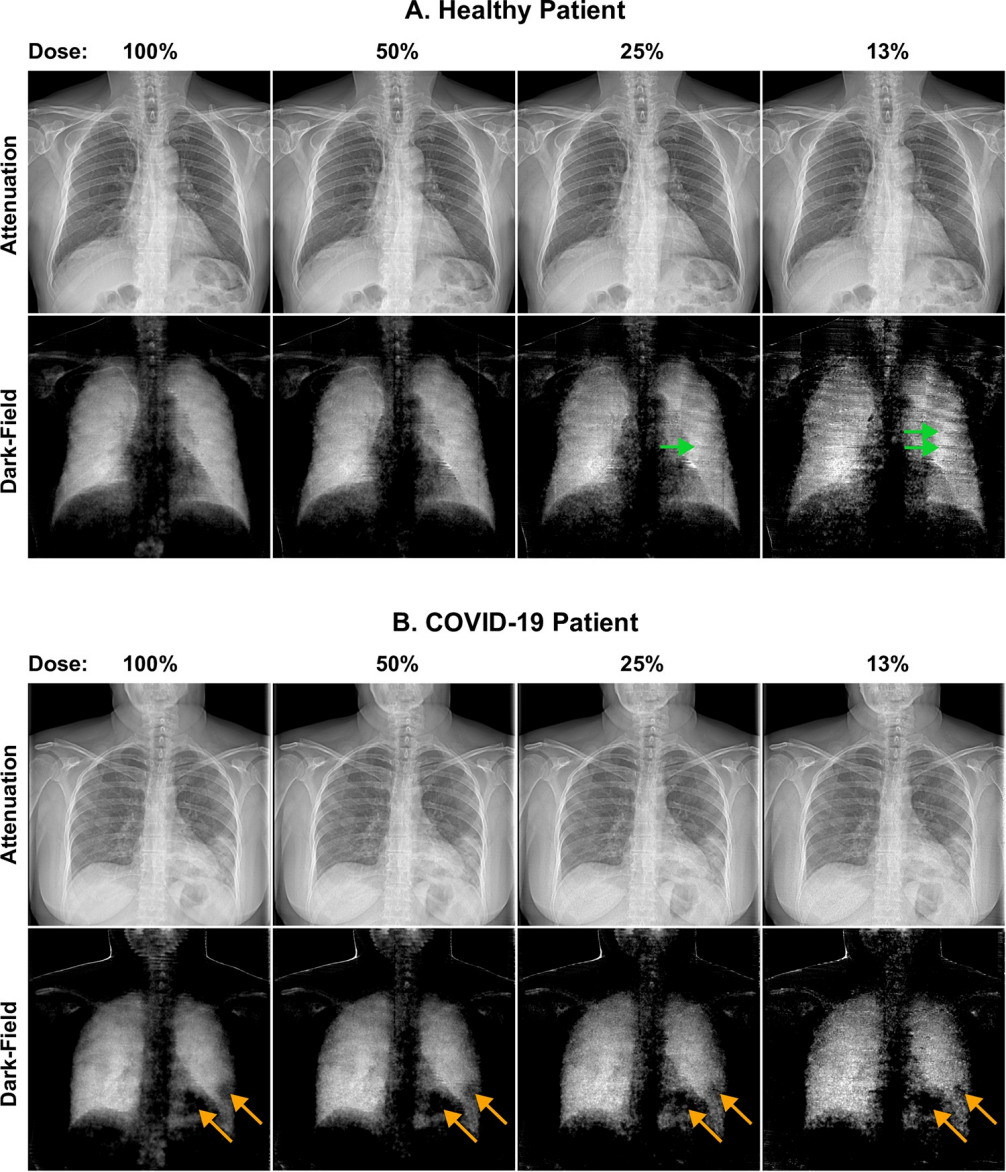
Attenuation and dark-field images at different dose levels. Attenuation and dark-field images of (A) an exemplary participant of the control group without lung disease (77 yrs., male) and (B) an exemplary COVID-19 participant with pneumonia (79 yrs., female) at the full dose of 100% and reduced simulated dose levels of 50%, 25% or 13%, respectively. Green arrows indicate stripe artifacts, best observed at the lowest simulated dose of 13%. Orange arrows indicate areas of focal signal loss (patchy pattern), typical for COVID-19 pneumonia observable at all dose levels.

Dark-field and attenuation images generated at the regular dose (i.e., 100%) and reconstructed at the simulated reduced dose levels (50%, 25%, 13%) are presented in Fig 2 for two exemplary study participants, one from the control group and one from the COVID-19 pneumonia group. At a simulated dose reduction of 50%, the dark-field images of both groups barely differ from the images obtained at the radiation dose of 100%. At a simulated radiation dose of 25% and particularly at a dose of 13%, however, the dark-field images may show streak artifacts (Fig 2A), which are to be attributed to the above-described dose reduction algorithm. Dark-field images of COVID-19 participants frequently reveal a patchy pattern (i.e., inhomogeneity of the dark field signal) and a signal loss in the affected lung areas (Fig 2B), both features typical for COVID-19 pneumonia, which are visible on the images at all simulated reduced radiation doses.

### 3.3. Reader study analysis

**Fig 3:**
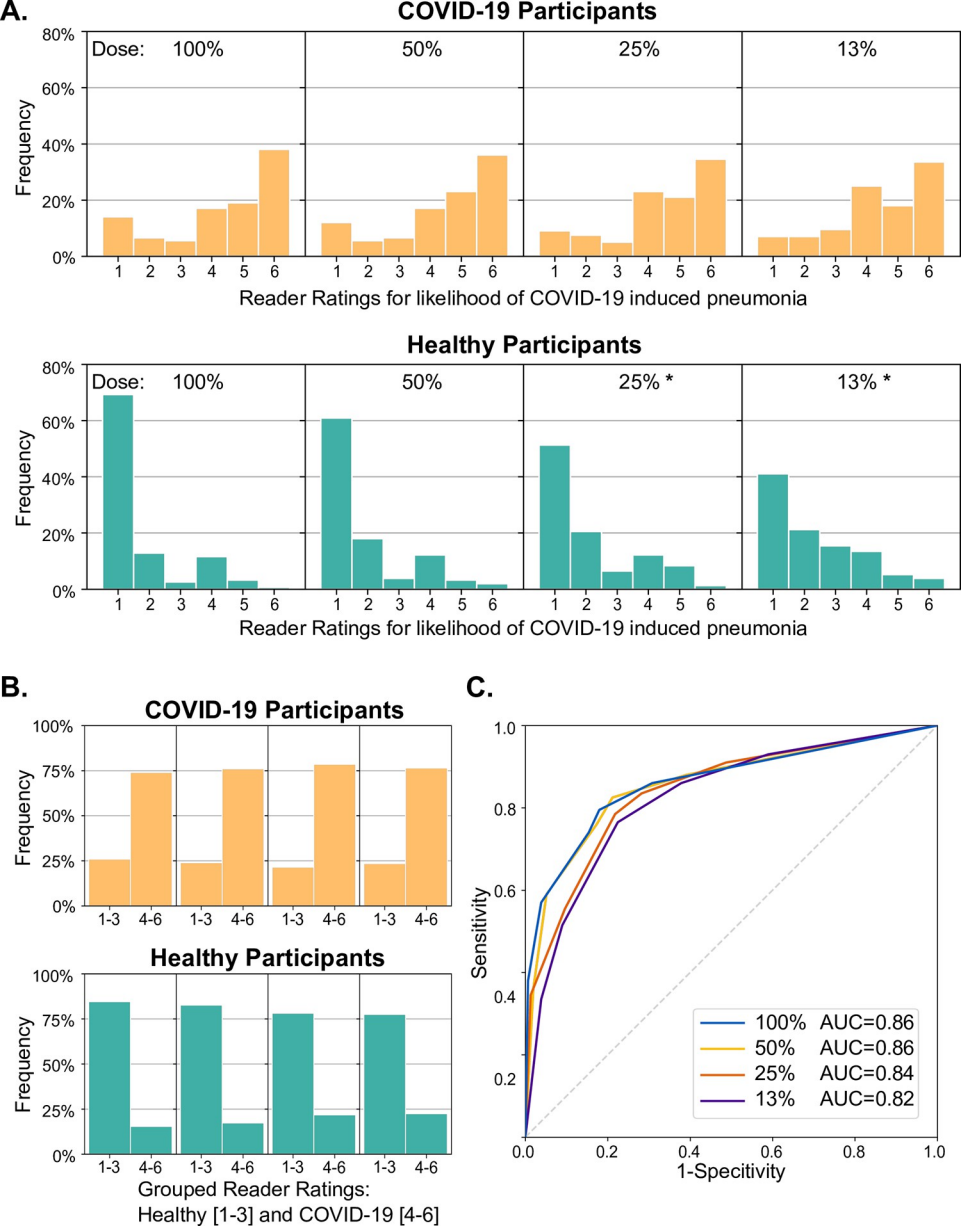
Reader study evaluation of the different dose levels. Frequency distributions of reader ratings, individual (A) and grouped (B), for likelihood of COVID-19-induced pneumonia for infected (orange) and lung-healthy (green) participants in combined dark-field and attenuation-based image readings. Significant differences (p<0.050) between the reader rating distributions compared to 100% of the dose are marked by asterisks (*). The receiver operating characteristics curves are shown in (C) for all dose levels. The area under their curve (AUC), which can be interpreted as the diagnostic performance, did not differ between the full dose (AUC = 0.86), 50% (AUC = 0.86 and p = 0.695), and 25% (AUC = 0.84 and p = 0.226) images and was slightly lower at 13% of the full dose (AUC = 0.82 and *p* = 0 .0378).

The ratings for the likelihood of COVID-19 induced pneumonia, as assessed by the readers’ side-by-side evaluation of dark-field and attenuation images are presented in Fig 3 for the different dose levels. When assessing full-dose images of participants with COVID-19, most readings for the presence of COVID-19 pneumonia were “6-surely” (38%), “5-very likely” (19%) or “4-likely” (17%). Virtual dose reduction to 50%, 25% or 13% did not change this distribution of reader ratings significantly (*p* = 0.744, *p* = 0.632, *p* = 0.632, respectively) (Fig 3A).

For participants of the control group, most readings for the presence of COVID-19 pneumonia were “1-surely not” (69%) or “2-very unlikely” (13%) (Fig 3A).

In contrast to the COVID-19 group, the distribution of reader ratings in the control group was significantly altered by virtual dose reduction to 25% and 13% of the full dose (*p*<0.001 in both cases), respectively (Fig 3A), with a significant decrease (*p*<0.001) in the reader-rating “1-surely not” (from 69% at full dose to 51% and 41% at the lowest simulated dose, respectively).

However, the grouped reader ratings for categorizing healthy participants (ratings 1 to 3) and COVID-19 participants (ratings 4 to 6) did not change with the simulated dose reduction (*p* = 0.734, *p* = 0.336, *p* = 0.644, respectively, for the healthy participants and the dose levels 50%, 25%, and 13%; *p* = 0.701, *p* = 0.121, and *p* = 0.071, respectively, for the COVID-19 participants) (see Fig 3B).

The inter-reader reliability as assessed by Krippendorff’s alpha coefficient was .60 for the full radiation dose of 100%, 0.57 for the 50% dose 0.56 for the 25% dose, and 0.46 for the 13% dose.

The readers’ performance for the detection of COVID-19 pneumonia at different radiation dose levels as assessed by ROC plots (Fig 3C) revealed AUC values of 0.86 for the full dose (95% CI: 0.81 to 0.91), of 0.86 for the 50% dose (95% CI: 0.81 to 0.91), of 0.84 for the 25% dose (95% CI: 0.79 to 0.89), and of 0.82 for the 13% dose (95% CI: 0.77 to 0.88) with only the latter being significantly lower than the 100% AUC value (*p* = 0.037; *p* = 0.695 and *p* = 0.226 for dose levels 50% and 12%, respectively).

These findings demonstrate that even at a simulated radiation dose of 25% the reader performance is comparable to that at full dose.

### 3.4. Quantitative analysis

**Fig 4:**
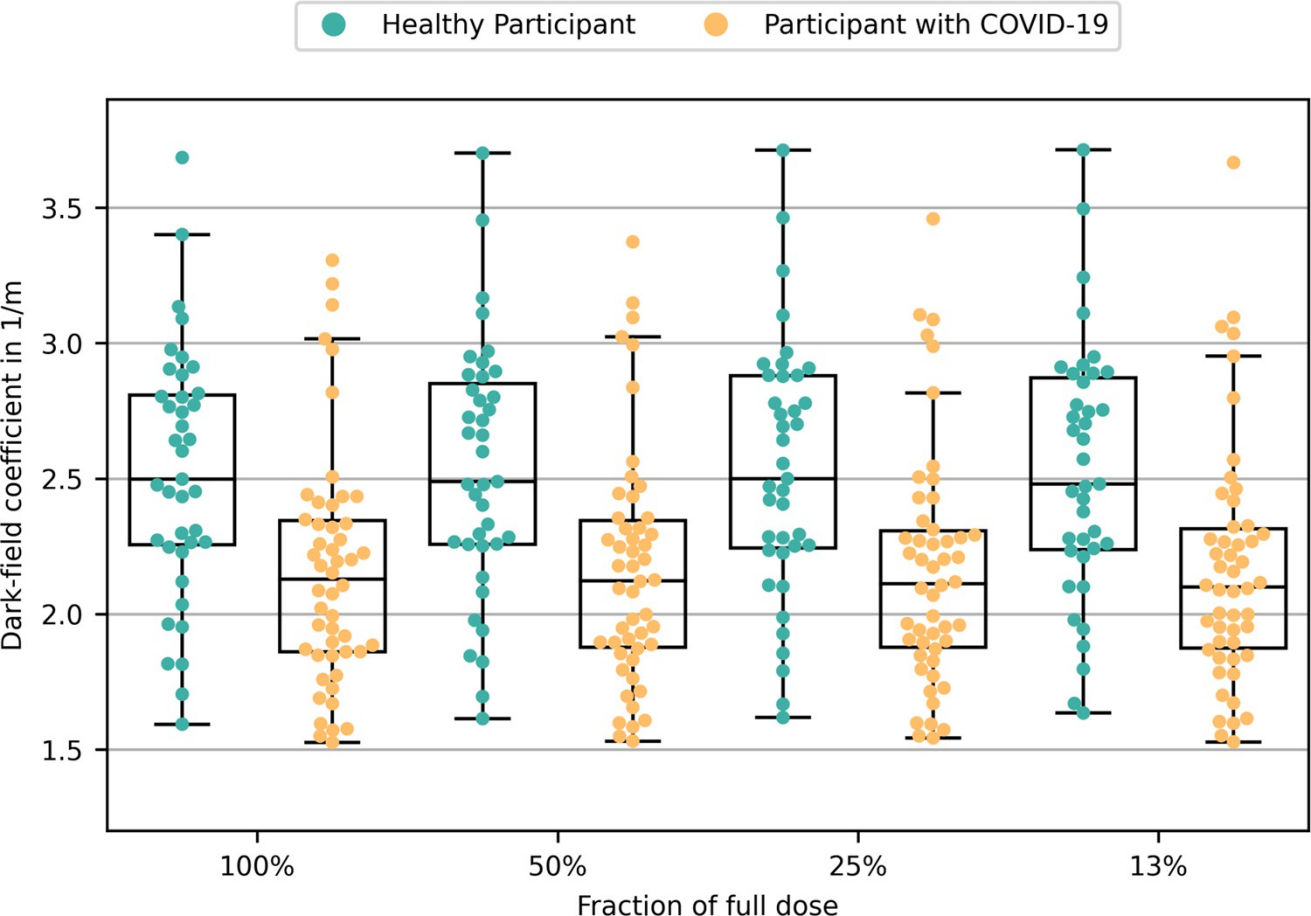
Quantitative evaluation of the different dose levels. Dark-field coefficients of control group participants (n = 39; controls; green dots) and COVID-19 participants (n = 50; orange dots) at different dose levels of 100% (full dose) and virtually reduced radiation doses of 50%, 25% and 13%, respectively. The distribution of dark-field coefficients of COVID-19 participants was significantly lower than the distribution of controls at each radiation dose examined (*p*<0.001).

In participants of the control group the median dark-field coefficient was 2.50 m^-1^ at 100% radiation dose which was not significantly altered by simulated dose reduction. In COVID-19 participants, however, the median dark-field coefficient was significantly reduced by 15% to 2.13 m^-1^ (*p*<0.001) (Fig 4 & Table 2), which was not altered by simulated dose reduction as well.

**Table 2:** Dark-field Coefficients (DFC) at different radiation doses.

Dose Level	DFC–Control	DFC–COVID-19
**100%**	2.50 (2.26 to 2.81)	2.13 (1.86 to 2.34)
**50%**	2.49 (2.26 to 2.85)	2.12 (1.88 to 2.34)
**25%**	2.50 (2.24 to 2.88)	2.11 (1.88 to 2.31)
**13%**	2.48 (2.24 to 2.87)	2.10 (1.88 to 2.31)

Dark-field coefficients for the control group and COVID-19 participants are given as medians (interquartile range).

The pairwise comparison of each participant’s dark-field coefficients obtained at the 50%, 25%, or 13% simulated dose levels with the dark-field coefficient of the full dose revealed differences. The 95th quantile of these differences was less than 2% (for the 50% dose), 5% (for the 25% dose), and 6% (for the 13% dose), respectively.

## 4. Discussion

The present study demonstrates that simulated dose reduction in dark-field chest radiography as low as 25% still allows for an excellent diagnostic performance in the assessment of COVID-19 pneumonia. This is of particular relevance since it has previously been shown that dark-field X-ray chest imaging enables a superior detection of COVID-19 pneumonia compared to conventional radiography and that combined use of both the attenuation and dark-field image modality outperforms each modality if employed independently [13].

While the effective radiation doses currently employed with the prototype X-ray scanning system for dark-field lung imaging are 1.75 times higher than those needed for conventional chest radiographs (20 μSv), the ALARA principle demands for lower radiation doses whenever feasible. To address the question whether COVID-19 pneumonia will remain detectable on dark-field images obtained with lower effective doses, as a first step, we determined the detectability of COVID-19 pneumonia on simulated dark-field chest images whose radiation dose was virtually reduced by an algorithm, that reconstructed the images from the raw intensity data with a virtually narrowed grating area’s height. Thus, this simulated height reduction of the grating area results in a change of the raw intensity data in such a way as if a real reduction of the radiation dose had been reached in the patient by using a narrower collimation. Therefore, a reduced area receiving the same x-ray intensity levels (as simulated by the algorithm) leads to the same total radiation dose as the full grating area receiving a reduced intensity level, which would be accomplished by real dose reduction. Moreover, with a real dose reduction (i.e. by a reduction of the X-ray tube current), algorithm-associated artifacts at very low doses (i.e. 25% and especially 13%) would be avoided and therefore even lower doses than the 25% might be applicable.

It should be noted that a reduction of the radiation dose is naturally associated with an increase in image noise. Such an increase of image noise will mostly appear in areas with low X-ray intensities at the detector, (e.g., in the abdomen, but not in the lung) and will attract readers’ attention only at lower simulated doses, particularly at the 13% dose.

In the present study, the AUC value of the ROC curve of readers’ diagnostic performance at the full dose was 0.86 (95% CI: .81 to 0.91) which is quite similar to the AUC value of 0.93 (95% CI: 0.91 to 0.96) previously reported by a readers’ evaluation of dark-field and attenuation radiographs [13]. This difference of the AUC values might originate from differences in study participants or a putative intra-reader variability. Nevertheless, the reader performance for the detection of COVID-19 in the present study lies within an excellent range (between 0.8 and 0.9) [27].

The ROC curves show that a simulated dose reduction of 50% and 25%, equaling median effective doses of 21.0 μSv and 10.5 μSv, respectively, has no significant (p > 0.050) impact on the diagnostic performance for assessing COVID-19 pneumonia. Dose reduction to 13% (= 5.4 μSv) also comes with a slightly reduced reader performance, (AUC = 0.82, *p* = 0.0374), which, however, can still be regarded as excellent.

While the dark-field coefficients at reduced doses remain similar to the dark-fieldcoefficients at the full dose within each group (control and COVID-19), the dark-field coefficients of COVID-19 participants are 15% lower than those of control group at all doses examined. This reflects a reduced number of air-tissue interfaces due to tissue inflammation (i.e. subsequent filling of the alveoli with inflammatory fluid in case of COVID-19 pneumonia) and is in line with previously reported data for the full dose [13].

A potentially small bias of the reader study might be due to the fact, that a negative COVID-19 PCR test was an exclusion criterion for the COVID-19 group, but a positive test was not mandatory for study inclusion. Further exclusion criteria were lung cancer, pneumothorax, and pleural effusion, but not other comorbidities such as COPD or pneumonia, which could have influenced the dark-field signal as well. However, this should not have affected the findings of our study towards the impact of dose reduction in dark-field radiography.

## 5. Conclusion

For the diagnosis of COVID-19 pneumonia, dark-field chest radiography with a simulated effective dose of 10.5 μSv (i.e., 25% of the regular dark-field dose) can be used without loss of diagnostic accuracy. A dose of 25% of the regular dark-field dose is even less than half the dose of conventional radiography, while providing additional image information.

To further assess the potential of low-dose dark-field chest radiography the dose reduction algorithm should be applied to other lung diseases. In conclusion, our results contribute an important step towards a significant reduction of the radiation dose in dark-field radiography without a relevant loss of diagnostic accuracy.

## Abbreviations

ROC: Receiver Operating Characteristic
AUC: Area Under the ROC Curve
DFC: Dark-Field Coefficient
CO-RADS: COVID-19 Reporting and Data System
Px: Pixel
RT-PCR: Reverse Transcription Polymerase Chain Reaction
CI: Confidence Interval
PACS: Picture Archiving and Communication Syste
